# The complete mitochondrial genome of the blue skate *Dipturus batis*

**DOI:** 10.1080/23802359.2020.1778572

**Published:** 2020-06-17

**Authors:** Aurélien Delaval, Tanja Schwanck, Martina Elisabeth Luise Kopp, Galice Hoarau, Catherine S Jones, Leslie R Noble

**Affiliations:** a Faculty of Biosciences and Aquaculture, Nord University, Bodø, Norway; b School of Biological Sciences, University of Aberdeen, Aberdeen, UK

**Keywords:** *Dipturus batis*, mitogenome, Rajidae

## Abstract

The complete mitochondrial genome of the blue skate *Dipturus batis* is described from shotgun sequencing on an Illumina next-generation sequencing platform. We report a 16,911 bp long sequence similar in size to other members of the genus, containing 13 protein-coding regions, 22 *tRNA* genes, 2 *rRNA* genes, and 2 non-coding areas. Phylogenetic analysis was performed using the complete mitochondrial genomes of 17 related species, placing *D. batis* within the Rajini tribe of the Rajidae family, consistent with current taxonomy. The new resource adds to a growing database of rajid mitogenomes which will help resolve phylogenetic relationships within the family.

The blue skate *Dipturus batis* (Family Rajidae, previously *Dipturus* cf. *flossada*) occurs on the continental shelf of the north-east Atlantic, primarily around the British Isles. *D. batis* has only recently received species status, after morphological and genetic investigations distinguished it from other large skates, particularly *D. intermedius* (Griffiths et al. 2010; Iglésias et al. 2010). Taxonomic confusion remains an issue among large skates, many of which are of conservation concern, and could benefit from additional tools.

We report the complete mitochondrial genome of *D. batis* from the fin clip (Natural History Museum London accession NHMUK014391967) of a male collected from the Rockall plateau (57°09′6′′N, 14°19′3W) on 10 September 2012 during a Marine Scotland Science survey. The individual was identified based on morphology including size of mature specimen, eye color, and dorsal patterning (Iglésias et al. 2010), and genetically (M. Frost pers comm). DNA was extracted using a DNeasy^®^ Blood & Tissue Kit (QIAGEN, Hilden, Germany) and shotgun sequenced on an Illumina^®^ NextSeq^®^ 500 using paired-end sequencing after library preparation using the NEBNext^®^ Ultra^™^ II DNA Library Prep Kit for Illumina (New England Biolabs^®^, Ipswich, MA, USA). The 32,180,856 raw reads generated were trimmed with BBDuk (30,920,799 reads remained) and mapped against the mitogenome of *D. oxyrinchus* (NC_037967) in Geneious version R9.1(Biomatters Ltd., Auckland, New Zealand). A total of 9077 reads mapped to the reference with 100% coverage (mean coverage depth 75.8), and produced a consensus sequence of 16,911 bp. To validate the consensus sequence we also performed a *de-novo* assembly using MINIA (kmer length 71), after trimming raw reads with TrimGalore and quality checking using FastQC, and BLASTed this assembly against the reference-mapped consensus sequence from Geneious.

The 16,911 bp consensus mitogenome (GenBank accession number MN820820) we report is similar in size to other members of the genus (16,907–16,913). The consensus was annotated using MitoAnnotator (Iwasaki et al. 2013) and MITOS (Bernt et al. 2013). Gene order and structure was typical of vertebrate mitogenomes and to that of *Dipturus* relatives, containing 13 protein-coding regions, 22 *tRNA* genes, 2 *rRNA* genes, and two non-coding areas (control region and the origin of L-strand replication). Nucleotide frequencies were: 30.1% A, 26.8% C, 14.3% G, 28.8% T (A + T composition of 58.9%). This is comparable to that reported for other *Dipturus* species (Vargas-Caro et al. 2016).

We performed a phylogenetic analysis in Geneious using the full mitochondrial genome sequences of 17 related species. Sequences were aligned using MAFFT and the phylogenetic tree was constructed using Bayesian inference, as implemented in the MrBayes (Huelsenbeck and Ronquist 2001) plugin, using the GTR substitution model for 500,000 iterations, sampling every 500th iteration after a burn-in of 100,000. *Atlantoraja castelnaui* (NC_025942.1) was used as the out-group. The phylogenetic tree places *D. batis* within the monophyletic clade of hardnosed skates (Rajidae), and within the tribe Rajini, consistent with current taxonomy (Last et al. 2016) (Figure 1). Rajidae currently includes 159 validated species (Fricke et al. 2019) within which re-assignments of genera are likely to occur (Last et al. 2016). The mitogenome of *D. batis* adds to a growing database that can be used to resolve phylogenetic relationships in the future.

**Figure 1.**
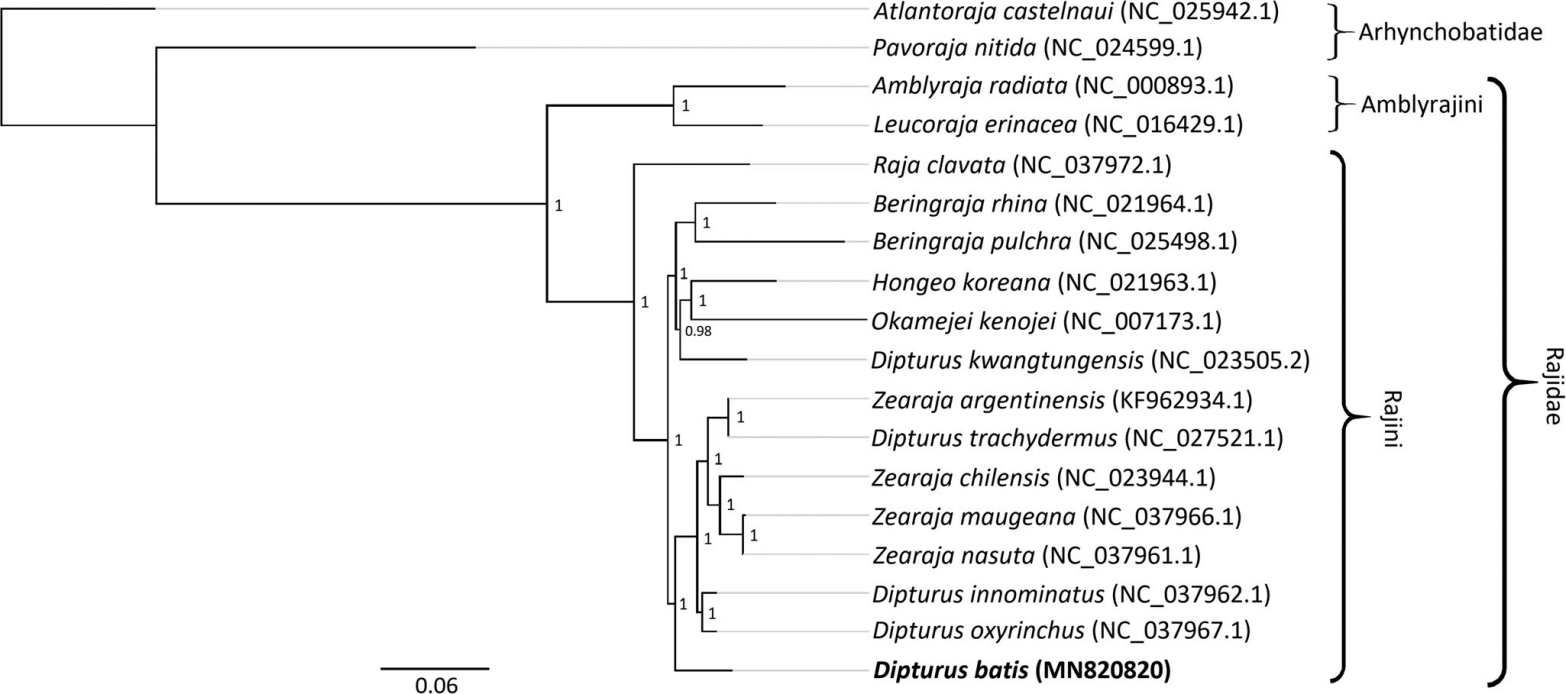
Phylogenetic tree of Dipturus batis (in bold) and 17 other species inferred from their complete mitogenomes. Scale bar shows the number of substitutions per site (thick black line), and posterior probabilities are shown for each node. Species names following Last et al. (2016) and GenBank accession numbers are given.

## Data Availability

The data that support the findings of this study are openly available in Genbank at https://www.ncbi.nlm.nih.gov/search/all/?term=MN820820, accession number MN820820.
